# A Pipeline to Determine RT-QPCR Control Genes for Evolutionary Studies: Application to Primate Gene Expression across Multiple Tissues

**DOI:** 10.1371/journal.pone.0012545

**Published:** 2010-09-02

**Authors:** Olivier Fedrigo, Lisa R. Warner, Adam D. Pfefferle, Courtney C. Babbitt, Peter Cruz-Gordillo, Gregory A. Wray

**Affiliations:** 1 Biology Department, Duke University, Durham, North Carolina, United States of America; 2 Institute for Genome Sciences & Policy, Duke University, Durham, North Carolina, United States of America; 3 Department of Evolutionary Anthropology, Duke University, Durham, North Carolina, United States of America; University of Texas Arlington, United States of America

## Abstract

Because many species-specific phenotypic differences are assumed to be caused by differential regulation of gene expression, many recent investigations have focused on measuring transcript abundance. Despite the availability of high-throughput platforms, quantitative real-time polymerase chain reaction (RT-QPCR) is often the method of choice because of its low cost and wider dynamic range. However, the accuracy of this technique heavily relies on the use of multiple valid control genes for normalization. We created a pipeline for choosing genes potentially useful as RT-QPCR control genes for measuring expression between human and chimpanzee samples across multiple tissues, using published microarrays and a measure of tissue-specificity. We identified 13 genes from the pipeline and from commonly used control genes: *ACTB*, *USP49*, *ARGHGEF2*, *GSK3A*, *TBP*, *SDHA*, *EIF2B2*, *GPDH*, *YWHAZ*, *HPTR1*, *RPL13A*, *HMBS*, and *EEF2*. We then tested these candidate genes and validated their expression stability across species. We established the rank order of the most preferable set of genes for single and combined tissues. Our results suggest that for at least three tissues (cerebral cortex, liver, and skeletal muscle), *EIF2B2*, *EEF2*, *HMBS*, and *SDHA* are useful genes for normalizing human and chimpanzee expression using RT-QPCR. Interestingly, other commonly used control genes, including *TBP*, *GAPDH*, and, especially *ACTB* do not perform as well. This pipeline could be easily adapted to other species for which expression data exist, providing taxonomically appropriate control genes for comparisons of gene expression among species.

## Introduction

Humans and chimpanzees are about 98.8% similar at the genomic level of alignable sequences [1]. Despite this modest genetic divergence, they vary in many remarkable behavioral and morphological aspects. Chimpanzee-human comparisons not only provide insights into human origins and contribute to understanding the evolution of uniquely human traits, they also provide practical medical insights; although there may be pathological and prognostic differences, the fact remains that chimpanzees and humans differ in susceptibility and outcomes for many diseases [2]. For instance, Alzheimer's disease is more common in humans and progression to AIDS is very rare in chimpanzees infected with HIV [3]. Humans and chimpanzees are almost identical at the protein sequence level; hence, it has been hypothesized that most of the phenotypic differences are caused by the regulation of gene expression [4]. Many studies have therefore focused on detecting differences in gene expression between these two species [5]–[16].

Gene transcript levels can be very precisely and reproducibly measured with quantitative real-time polymerase chain reaction (RT-QPCR). This technique is a relatively inexpensive technology for assaying the expression of a small number of genes. RT-QPCR is often preferentially used because of its wider dynamic range, compared to that of microarrays [17], and is used for corroborating results obtained from deep RNA sequencing [18]. However, the accuracy of RT-QPCR can be confounded by many sources of variation, including the total RNA content of the sample, the number of cells in the starting material, the RNA extraction efficiency, differential enzymatic efficiencies, and transcriptional activity [19].

One of the most widely used approaches to correct for these variables is the normalization of expression levels with control genes [20]. These control genes, also called normalizers or reference genes, are often chosen from “housekeeping” genes because they are expected to be evenly expressed across most tissues and samples. However, caution is required when choosing control genes. In particular, control genes that are not equally expressed across samples, especially from different tissues or species, can affect the accuracy of the calculation of relative expression differences between samples. Moreover, it has been shown that using only a single control gene can lead to appreciable normalization errors and that using several normalizers is preferable in order to compensate for the potential biases introduced by an inappropriate normalizer [19], [21]–[22]. Despite these known pitfalls, the choice of control genes for RT-QPCR is usually dictated by customary usage rather than empirical evidence and, most often, only a single control gene is used [23] (e.g. [7], [24]). It seems more and more evident that finding “universal” RT-QPCR control genes is a nearly impossible task. Instead, it is necessary to identify control genes that are most appropriate for the species, experimental conditions, and tissue types being assayed [20]–[21], [25]–[30].

In order to find control genes for comparing human-chimpanzee gene expression across multiple tissues, we developed a pipeline that draws on information from published microarray datasets for identifying candidate normalizers. These genes were chosen according to several criteria, including low variance within and between species and equal expression across tissues. We then validated each of the candidate control genes and proposed a minimal set of empirically validated control genes appropriate for assaying transcript abundance in cerebral cortex, liver, and skeletal muscle genes.

## Results and Discussion

In order to understand the genetic basis of many human specific traits, it is often relevant to ask whether particular genes are differentially expressed between humans and their closest living relative, the chimpanzee [4]. For studies involving a small number of genes, RT-QPCR is the gold standard for assaying transcript abundance as it presents several practical advantages [17]. However, this technology requires the use of control genes for normalization across multiple samples to account for technical and intrinsic variation [20]. Ideally, these control genes should be constantly expressed across all assayed samples. In the case of inter-species comparisons, the selection of these genes is particularly challenging since they need to be steadily expressed at multiple levels of comparison: within and between species as well as across tissues. Because “universal” control genes probably do not exist, it is important to identify appropriate genes for each project [20]–[21], [25]–[30]. In order to find such genes for comparing human and chimpanzee gene expression across multiple tissues, we developed a pipeline that consists of three steps: (1) determine a set of genes from published microarray studies with low variation between and within species as well as across tissues; (2) design and test for specificity primers for these genes; and (3) perform expression assays and variation analyses to determine the best set of control genes.

### Candidate reference genes

We computed the evenness score [31] for 22,667 genes from the Novartis expression atlas for 27 human tissues and examined within and between human-chimpanzee variation for 4,365 genes and five tissues. We were able to calculate combined variation scores (see Materials and Methods) for 3,556 genes present in both the Novartis expression atlas and the human-chimpanzee microarray dataset (Table S1). We were interested in the top 5% of the list (∼178 genes) with the smallest score. Among these promising genes, we selected five genes with non-related functions and spanning a range of expression levels: *GSK3A*, *USP49*, *EEF2*, *ARHGEF2*, and *EIF2B2* (Table 1). For comparison, we selected commonly used control genes in the literature: *ACTB*, *GAPDH*, *HMBS*, *HPTR1*, *RPLI3A*, *SDHA*, *TBP*, and *YWHAZ* (Table 1). These genes have been used in numerous studies; *GAPDH* and *ACTB*, in particular, have been used in previous comparative primate gene expression studies [7], [24]. Interestingly, *ACTB*, *GAPDH*, and *TBP* were not among the 5% most stable genes (Table 2). *HMBS*, *SDHA*, and *RPL13A* were not present in the primate microarray dataset. *HMBS* and *SDHA* were respectively within the 15 and 50% most stably expressed genes across the 26 Novartis tissues. Due to a lack of correlation between transcript abundance measurements from microarray and RT-QPCR technologies, our method does not guarantee that optimal genes identified in this step of our pipeline are going to be the most optimal genes for RT-QPCR. However, we believe that the top 5% of the list of candidates contains genes with desirable properties for further testing with the geNorm method.

**Table 1:** List of candidate genes.

Symbol	Name	Function	Rank
*GSK3A*	Glycogen synthase kinase 3 alpha	Involved in hormonal control of regulatory proteins	13*
*USP49*	Ubiquitin specific peptidase 49	Breakdown peptides	15*
*EIF2B2*	Eukaryotic translation initiation factor 2B, subunit 2 beta	Involved in protein synthesis	42*
*ARHGEF2*	Rho/rac guanine nucleotide exchange factor (GEF) 2	Activates Rho GTPases, involved in numerous cellular processes initiated by extracellular stimuli (cell cycle, motility, barrier etc…)	56*
*EEF2*	Eukaryotic translation elongation factor 2	Essential factor for protein synthesis	162*
*ACTB*	Beta actin	Cytoskeletal structural protein	450
*TBP*	TATA box binding protein	RNA polymerase II transcription factor	761
*GAPDH*	Glyceraldehyde-3-phosphate dehydrogenase	Glycolytic enzyme	990
*YWHAZ*	Tyrosine 3-monooxygenase/trytophan 5-monooxygenase activation protein, zeta polypeptide	Mediate signal transduction by binding to phosphoserine-containing proteins	2302
*HPRT1*	Hypoxanthine phosphoribosyl-transferase 1	Generation of purine nucleotide through the purine salvage pathway	2346
*HMBS*	Hydroxymethylbilane synthase	Heme synthesis and porphyrin metabolism	?
*SDHA*	Succinate dehydrogenase complex, subunit A	Transfer electrons in the TCA cycle and respiratory chain	?
*RPL13A*	Ribosomal protein L13a	Structural constituent of ribosome	?

Genes are ranked according to variation between/within species and evenness across 25 tissues.

*Indicates a gene among the top 5%.

**Table 2:** Primer sequences for candidate control genes, efficiency, and primerBlast results.

Symbol	Forward primer	Reverse Primer	Eff%	PrimerBlast Hit(s)	Chr#
*GSK3A*	CCCAACTACACGGAGTTCAA	CCAGCAGGCTAGAGCAGAG	92.2	*GSK3A*	19*
*USP49*	CTCAGCCACCTCCAGAAGTT	AAAGCTGAGTCTTCCCGTTG	95.8	*USP49*	6*
*EIF2B2*	TCAAGATTATCCGGGAGGAG	ATGGAAGCTGAAATCCTCGT	96.5	*EIF2B2*	14*
*ARHGEF2*	ATCTACCCCTCCGACAGCTT	CCAGGGGAGACTCATCATTG	95	*ARHGEF2*	1*
*EEF2*	AGAAGCTGTGGGGTGACAG	GATCAGCTGGCAGAAGGTG	96.2	*EEF2*	19*
*ACTB*	CTGGAACGGTGAAGGTGACA	AAGGGACTTCCTGTAACAATGCA	**109.4**	*LOC644936 (Beta-actin pseudogene)*	5*
				*ACTB*	7
				*A26C1B*	2
				*LOC653269*	2
*TBP*	GCTGAGAAGAGTGTGCTGGA	GTAAGGTGGCAGGCTGTTGT	95.8	*TBP*	6*
*HMBS*	GGCAATGCGGCTGCAA	GGGTACCCACGCGAATCAC	99.2	*HMBS transcript variant 1*	11*
*SDHA*	TGGGAACAAGAGGGCATCTG	CCACCACTGCATCAAATTCATG	99.6	*SDHA*	5*
				*SDHALP1*	3
				*SDHALP2*	3
				*LOC220729 (SDHA pseudogene)*	3
				*LOC100134106*	3

*Indicates that both the forward and reverse primers are perfect matches.

### Primer design and efficiencies

**Figure 1:**
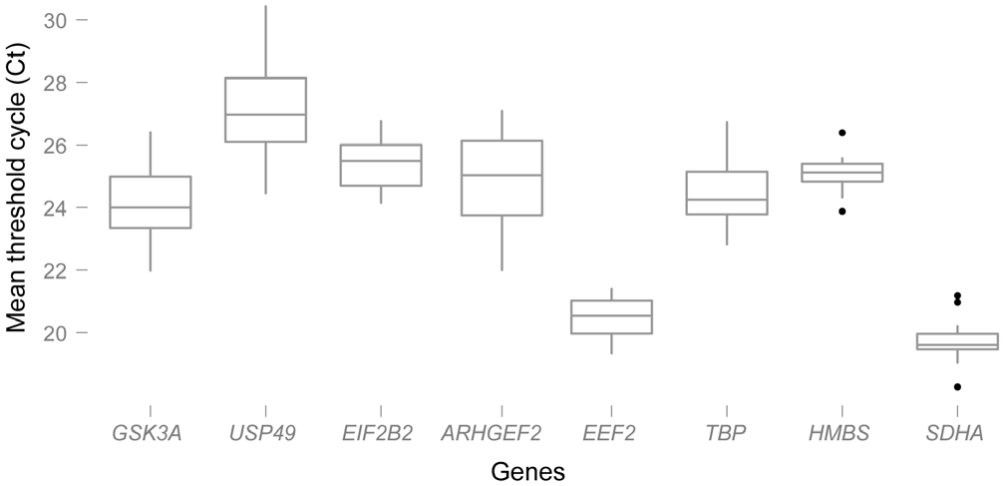
Variation of threshold cycle levels. Box plot of threshold cycle levels of candidate genes for human-chimpanzee samples across combined tissues: skeletal muscle, liver, and cerebral cortex.

We identified a list of genes for further testing, comprising the five new candidates and four commonly used control genes: (*GSK3A*, *USP49*, *EEF2*, *ARHGEF2*, and *EIF2B2*) and (*ACTB*, *SDHA*, *HMBS*, and *TBP*). We used published primer sets for three of the commonly used genes (*ACTB*, *HMBS*, and *SDHA*) [19], and we designed new primer sets for the remaining six genes. We tested these primers and estimated their efficiencies using cDNA from the IMR32 cell line (Table 2). Interestingly, the published primer set of *ACTB* had an efficiency greater than 100% and a Primer-Blast analysis resulted in multiple hits, the best hit not being *ACTB*. We therefore did not include this gene in subsequent analyses. “Primer-Blasting” *SDHA* primer sets also lead to multiple hits. However, the fact that the best hit perfectly mapped onto *SDHA* and the fact that the primer efficiency was below 100%, did not suggest multiple non-specific amplifications. All the other primer sets had an efficiency ranging from 92.2% to 99.6% and a unique sequence match to the appropriate gene in both the human and chimpanzee genomes. We then narrowed our list to eight genes: *GSK3A*, *USP49*, *EEF2*, *ARHGEF2*, *EIF2B2*, *SDHA*, *HMBS*, and *TBP*. For all eight genes, the threshold cycle values (Ct) vary between 19.42 and 30.5 (Figure 1). When tissues and species are combined, *HMBS*, *SDHA*, *EEF2*, and *EIF2B2* show the least Ct variation, while *TBP* and *USP49* show the largest variation.

### Expression stability

**Figure 2:**
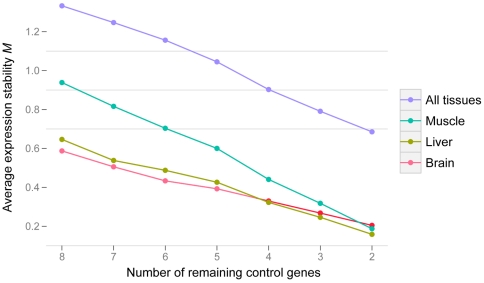
Average expression stability with iterative exclusion of the least stable gene. Low average *M* values indicate high stability and high average *M* values indicate less stability.

**Figure 3:**
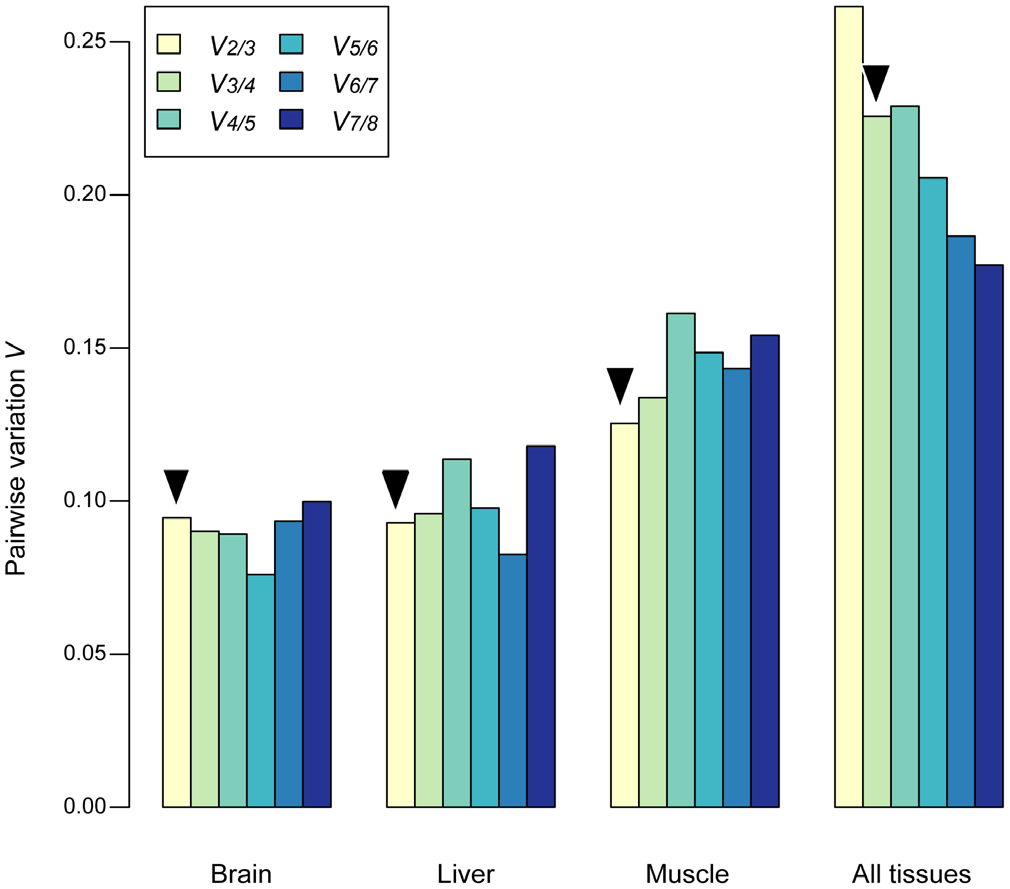
Optimal number of control genes. The optimal number of control genes was determined by pairwise variations (*V_n/n+1_*) between the normalization factors *NF_n_* and *NF_n+1_*. The black arrows indicate the optimal number of genes to use for RT-QPCR normalization. For each tissue, the inclusion of a fourth gene does not significantly change the normalization factor. For all tissues combined, the use of a fourth gene has a large effect on the normalization factor.

In order to determine the most stably expressed genes from the list of eight candidate genes, we used the geNorm method developed by Vandesompele et al. [19]. We measured expression levels across three tissues (liver, cerebral cortex, skeletal muscle) for all eight genes for humans and chimpanzees (two individuals per species). The eight genes were ranked according to their stability score *M* for each tissue and for all tissues combined. Iteratively, the gene with the highest score (largest variability) was excluded until we reached the last gene pair (Figure 2; Table 3). Liver and cerebral cortex tissues exhibited more stability than skeletal muscle overall, but they converged to the same average stability score when all genes were included. As expected, there is more variation in all tissues combined than in each tissue separately, even when we included all reference genes. Considering tissues taken individually or combined, including all eight genes seems to be the optimal strategy. However, there is a tradeoff between optimality and practicality. In order to determine the minimal set of genes for which stability would be acceptable and practical, we calculated and plotted a variation coefficient for including an additional gene (Figure 3; Table 3). If we consider the tissues separately (cerebral cortex, skeletal muscle and liver), the best three normalizers are respectively (Table 3): (*EIF2B2*, *HMBS*, *SDHA*), (*HMBS*, *EEF2*, *GSK3A*), and (*EIF2B2*, *USP49*, *TBP*). Adding a fourth gene does not drastically affect the normalization factor. However, as expected, if we consider all tissues together, the inclusion of a fourth gene has a large effect on the calculation of the normalization factor (*HMBS*, *EEF2*, *EIF2B2*, and *SDHA*). As practical limitations often preclude the use of too many genes, we recommend using at least two from this quartet whose Ct range is close to that of the target genes. The goal of our study is to determine appropriate genes for multiple species and across several tissues. The observed discrepancy between individual tissues and all tissues in table 3 reflects both a bias produced by the way we selected candidate genes from microarray datasets and the difficulty of finding “universal” control genes. Finding optimal control genes for multiple tissues does not guarantee that they are the best for individual tissues. If one wants to focus on a single tissue, it is preferable to determine a new set of candidate genes for this tissue alone.

**Table 3:** Ranking of candidate control genes after geNorm analyses for individual and combined tissues.

All tissues	Cortex	Muscle	Liver
*EIF2B2-EEF2*	*EIF2B2-HMBS*	*EEF2-GSK3A*	*EIF2B2-USP49*
*HMBS*	*SDHA*	*HMBS*	*TBP*
*SDHA*	*GSK3A*	*ARHGEF2*	*HMBS*
*TBP*	*EEF2*	*EIF2B2*	*GSK3A*
*GSK3A*	*TBP*	*TBP*	*SDHA*
*ARHGEF2*	*ARHGEF2*	*SDHA*	*EEF2*
*USP49*	*USP49*	*USP49*	*ARHGEF2*

The best pair of genes is listed first.

### Conclusion

In addition to proposing, for the first time, a set of adequate reference genes for comparing human and chimpanzee gene expression, our study also proposes a pipeline that can be easily adapted and applied to other tissue and species comparisons. Many control gene lists have been previously published [28], [32]–[35] but they are limited to their own specific application. In addition, our approach is not based entirely on an *a priori* candidate gene list but also includes genes based on comparative studies across multiple species and tissues using a novel calculation of tissue expression evenness [31]. While other studies have proposed methods to detect candidate control genes based on microarray data [23], [34]–[35], to our knowledge, our pipeline is the first attempt to implement an approach appropriate for comparisons among species.

## Materials and Methods

### Gene selection

An appropriate control gene for comparing human and chimpanzee expression across multiple tissues was defined by steady expression between and within species as well as across tissues. We established a genome-wide list of candidate normalizers by comparing their level of expression from published microarray studies. Specifically, we used human microarray data from the Novartis Gene Expression Atlas (http://biogps.gnf.org/) and several microarrays from a human-chimpanzee study [14].

First, the Novartis expression dataset was analyzed to assess human gene expression evenness across tissues. We examined 22,667 genes in 26 selected non-cancerous tissues: central nervous system (temporal lobe, globus pallidus, cerebellum peduncles, cerebellum, caudate nucleus, whole brain, parietal lobe, medulla oblongata, amygdala, prefrontal cortex, occipital lobe, thalamus, subthalamic nucleus, cingulate cortex, pons, fetal brain, olfactory bulb), skeletal muscle, kidney, liver, heart, and testis (testis, testis leydig cell, testis germ cell, testis interstitial, testis seminiferous tubule). We determined the evenness of expression across these tissues for each gene based on a previously published approach [31]. Briefly, a gene can be plotted as a vector in a multi-dimensional space representing its expression in every tissue (i.e. each tissue is represented by an axis). We used a geometric calculation to determine the evenness of expression across all tissues. If a gene were perfectly evenly expressed, its vector would form equal angles with each axis – we defined this as the expected expression vector. The evenness score *e* of a gene is calculated as the squared cosine of the angle ε between the actual expression vector and the expected expression vector: 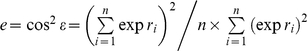 where *n* is the total number of assayed tissues and *expr_i_* is the gene expression for tissue *i*. If this gene is equally expressed across all tissues, *e* = 1, while, if it is only expressed in a single tissue, *e* will be small (0.04 in the case of 25 tissues). We then ranked all genes according to their expression evenness.

Secondly, we used the human-chimpanzee microarray dataset (11,780 genes) [14] to determine whether a gene has constant expression within and between species. For the genes for which both within and between species variation has been assayed as the mean squared difference (4,365 genes), we calculated and normalized the average and standard deviation within and between species for all five tissues (brain, heart, testis, kidney, and liver) [14]. The goal of this step was to identify genes that have small differences between and within species across the five tissues. We sorted the list of genes according to the product of these values (score of differences). Finally, we intersected the gene lists established from the evenness calculation and from the human-chimpanzee microarrays and calculated a combined variation score (product of 1-evenness and score of differences). We then ranked them from the most steady (low score) to the least steady (high score). Although outliers may influence it, this metric is sufficient for establishing a list of candidates for further scrutiny (i.e. geNorm analysis). We chose a final set of candidate genes from among the high scoring genes (top 5%) according to three additional criteria: (1) genes that cover a wide range of expression levels and for which the combined variation score is not mainly influenced by one extreme value; (2) genes with a “housekeeping” function which may be more likely to be valid control genes; and (3) genes that are unlikely to be co-regulated. For comparison, we also included a few commonly used control genes.

### RT-QPCR

Human total RNA samples were obtained from Biochain (http://www.biochain.com/) and chimpanzee tissue samples from the Southwest Foundation for Biomedical Research for cerebral cortex (humans: A803159, A803148; chimpanzees: 4X0505, 4X0391), liver (humans: A602084, A507018; chimpanzees: 4X0505, 4X0391), and skeletal muscle (humans: A811244, A508352; chimpanzees: 4X0505, 4X0391). Different RNA extraction kits and protocols were used for processing different tissues: QIAGEN RNeasy® Lipid Tissue Kit for cerebral cortex samples, QIAGEN RNeasy® Kit for liver samples, and QIAGEN RNeasy® Fibrous Tissue Kit for skeletal muscle samples. 6µg of total RNA were reverse transcribed into cDNA according to the manufacturer's protocol using a High Capacity cDNA Reverse Transcription Kit Archive Kit (P/N 4368813) from Applied Biosystems®. For all candidate genes, primers were designed in conserved exonic regions across species and transcript isoforms. Sequences of these regions were then mapped to the human and chimpanzee genomes to verify their uniqueness using Primer-Blast (http://www.ncbi.nlm.nih.gov/tools/primer-blast/). Primers were designed using Primer3 [36] with the following parameters: 100–150 base pairs (bp) product long, primer Tm min 58°C, opt 59°C, max 61°C; primer size min 17 bp, opt 20 bp; max 23 bp. We performed standard curves on each set of primers using cDNA from an IMR32 cell line with a dilution series (eight dilutions). The R^2^ of this dilution series and primer set efficiency were calculated. The efficiency was determined as follows: 
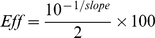
 where *slope* is the regression line slope. Expression levels were measured on the ABI PRISM 7000. 30 µl reactions contained 15 µl 2× ABGene Absolute q-PCR SYBR® Green Mix, 0.75 µl for each primer (10 µM), 1 µl of cDNA template, and PCR quality water to reach the desired volume. The RT-QPCR program was: 95°C for 15 minutes, 40 cycles of a 15 second melt at 95°C, and a 30 second annealing/elongation at 60°C. The program ended with a dissociation curve from 50 to 90°C. Reactions were done in technical triplicates and those with a standard deviation above 0.3 were excluded and rerun.

### Data analysis

We used the geNorm algorithm [19] implemented in the R package SLRT-QPCR (http://www.bioconductor.org/packages/2.2/bioc/html/SLRT-QPCR.html) and customized python scripts to determine the best set of control genes among all the candidates. The expression ratio of two ideal control genes should be similar across all samples, therefore the variation of these ratios should be small. To quantify the performance of a gene as an appropriate control gene, we used a gene-stability score *M*, that is the average pairwise variation of this gene with all the other genes. First, we calculated the relative quantity of each gene *g* and sample *i* with the delta-Ct formula [37]. Second, we assigned a stability rank to each gene using an iterative process: starting from the complete set of candidate control genes, we computed the stability measurement *M* for each gene and iteratively excluded the least stable (the gene with the lowest *M* is the most stable while the gene with the highest *M* is the least stable). Finally, we used this ranked list to determine the optimal set of genes for RT-QPCR normalization by calculating the effect of including one additional gene to the set [19]. We calculated a normalization factor *NF* (geometric mean of expression values) for subsets *n* and *n+1*; a large pairwise variation (*V_n/n+1_*) between *NF_n_* and *NF_n+1_* values indicates a non-negligible improvement for the inclusion of an additional gene.

## Supporting Information

Table S1 Complete results from the candidate control genes selection pipeline.
(0.94 MB XLS)
